# Supplementary material for: Clinical Utility of LCT Genotyping in Children with Suspected Functional Gastrointestinal Disorder
Source: Nutrients. 2020 Oct 1;12(10):3017. doi: 10.3390/nu12103017 (PMC7601291; doi:10.3390/nu12103017)
Supplement: Supplementary file 1 [file nutrients-12-03017-s001.pdf]

Table S1. Comparison of genotypes according to lactose absorption/malabsorption and lactose tolerance/intolerance.

	p-value (adjusted Fisher)								
HBT	Sample A (n = 493)			Sample B (n = 120)					
	C/T-13910 polymorphism			C/T-13910 polymorphism			G/A-22018 polymorphism		
	CC: CT	CC: TT	CT: TT	CC: CT	CC: TT	CT: TT	GG: GA	GG: AA	GA: AA
**Absorption/malabsorption**	$2.75e^{-62}$	$1.09e^{-34}$	$6.98e^{-01}$	2.78e-^{13}	9.23e-^{07}	2.84e-^{01}	$1.08e^{-12}$	$6.80e^{-07}$	1.37e-^{01}
**Tolerance/intolerance**	$9.81e^{-10}$	$3.04e^{-02}$	$3.35e^{-02}$	0.009	0.642	0.646	0.00348	0.63300	0.65000

In the case of the C/T-13910 polymorphism, analyses were performed for both samples (A and B). For the G/A-22018 polymorphism, the analysis was performed only for sample B. Abbreviation: HBT, hydrogen breath-test.
